# Learning from the emergence of NIHR Collaborations for Leadership in Applied Health Research and Care (CLAHRCs): a systematic review of evaluations

**DOI:** 10.1186/s13012-018-0805-y

**Published:** 2018-08-15

**Authors:** Roman Kislov, Paul M. Wilson, Sarah Knowles, Ruth Boaden

**Affiliations:** 1 Alliance Manchester Business School, University of Manchester, Manchester, UK; 2 NIHR Collaboration for Leadership in Applied Health Research and Care Greater Manchester, Manchester, UK

**Keywords:** CLAHRC, Evaluation, Collaboration, Learning health systems, Co-production, Knowledge mobilisation, Implementation

## Abstract

**Background:**

Collaborations for Leadership in Applied Health Research and Care (CLAHRCs) were funded by NIHR in England in 2008 and 2014 as partnerships between universities and surrounding health service organisations, focused on improving the quality of healthcare through the conduct and application of applied health research. The aim of this review is to synthesise learning from evaluations of the CLAHRCs.

**Methods:**

Fifteen databases including CINAHL, MEDLINE, EMBASE and PsycINFO were searched to identify any evaluations of CLAHRCs. Current and archived CLAHRC websites and the reference lists of retrieved articles were scanned to identify any additional evaluations. Searches were restricted to English language only. Any publications from evaluations of the CLAHRCs were eligible for inclusion if they fulfilled at least one of three pre-specified inclusion criteria. A narrative synthesis was undertaken.

**Results:**

Twenty-six evaluations (reported in 37 papers) were deemed eligible for inclusion. Evaluations focused on describing and exploring the formative partnerships, vision, values, structures and processes of CLAHRCs; the nature and role of boundaries; the deployment of knowledge brokers and hybrid roles to support knowledge mobilisation; patient and public involvement; and capacity building. The relative lack of data about the early impact of CLAHRCs on health care provision or outcomes is notable.

**Conclusions:**

Much of the evaluative focus on CLAHRCs has been on how they have been organised and on the development of theory around their emergent properties. Evidence is lacking on the impact of CLAHRCs particularly in relation to the knowledge mobilisation processes and practices adopted. Further evaluation of CLAHRCs and other similar research and practice partnerships is warranted and should focus on which knowledge mobilisation approaches work where, how and why.

**Trial registration:**

PROSPERO (Registration number: CRD42016042945).

**Electronic supplementary material:**

The online version of this article (10.1186/s13012-018-0805-y) contains supplementary material, which is available to authorized users.

## Introduction

Healthcare has long seen significant investment in the production of research evidence to inform decisions and choices around the delivery and organisation of services. However, making use of research-based knowledge routinely has been a challenge and one that has been described as the ‘second translation gap’ [1]. Growing recognition of the need to accelerate the generation and uptake of knowledge in health systems has led to a focus on the development of new models of research and practice partnership [2, 3]. Such collective knowledge mobilisation processes are increasingly viewed as integral to the development of learning health systems which seek to improve care through a continuous cycle of knowledge production and implementation [4].

In the USA, the Veterans Health Administration through its Health Services Research and Development Service and the Quality Enhancement Research Initiative has been at the forefront of efforts to enhance partnered research [5, 6]. This has been mirrored in other geographical settings, such as the establishment of Advanced Health Research and Translation Centres by the National Health and Medical Research Council in Australia [7]. In the UK, a report by the Chief Medical Officer’s Clinical Effectiveness Group in 2007 recommended that the National Health Service (NHS) should better utilise higher education to support initiatives to enhance the uptake of applied health research into routine practice [8]. This recommendation prompted the development of new models of research and practice partnership. In 2008, the National Institute for Health Research (NIHR) funded nine ‘pilot’ Collaborations for Leadership in Applied Health Research and Care (CLAHRCs): collaborative partnerships between universities and surrounding NHS organisations, focused on improving patient outcomes through the conduct and application of applied health research. Each CLAHRC was required to obtain ‘matched funding’ from partners to the value of the NIHR investment. The aim was to create and embed approaches to research and its application that are specifically designed to take account of the way that health care is delivered across sectors and a clearly defined geographical area.

In 2014, a further round of funding was awarded to 13 CLAHRCs across England with the same matched funding requirements [9]. Each CLAHRC has developed independently within a local context with key service stakeholders and researchers playing an important role in shaping the focus for research and improvement. The CLAHRCs therefore represent an ongoing nationwide experiment to improve collaboration between academic and health partners, and consequently to increase research impact for the benefit of patients.

In 2010, the NIHR Service Delivery and Organisation Programme (now known as the Health Services and Delivery Research (HS&DR) Programme) commissioned independent longitudinal research evaluations of the pilot CLAHRCs through an open call [10]. The call asked for evaluations that reflected ‘the dynamics, processes, emergent properties and diverse impacts of the CLAHRCs’ as they developed [10]. Applications that drew on the ‘broad diversity of evaluation approaches including exploratory, descriptive, experimental, programme and economic evaluation approaches’ were to be encouraged. The call also indicated that funded evaluations were expected to contribute to the growing international knowledge base on research use and impact and to generate evidence with broader applicability for the development of other research and practice partnerships beyond the CLAHRCs.

There have been no such evaluations commissioned by NIHR since these in 2010, and given that the second round of CLAHRC funding was not referred as ‘pilot’ funding, it might be assumed that NIHR have been convinced of the ‘value’ of CLAHRCs through the pilot funding round. None of the commissioned evaluations published their final reports before the second round of funding although it is possible that unpublished early findings were fed in informally to NIHR as part of the commissioning process for the 2014 funding round.

NIHR also required routine performance information from CLAHRCs, which was focused on research metrics used for other types of NIHR funding (e.g. biomedical research). These metrics included numbers of publications, numbers of funded students awarded higher degrees, additional research funding leveraged, impact on health care and patients through ‘case studies’ [10].

Our aim with this review is to synthesise what has been learnt through evaluation (and published) about the process and impact of the CLAHRCs. We have focused on published papers because of the requirement from the funded evaluations, and of CLAHRCs generally, to contribute to knowledge. Specifically, we are interested in what evaluations tell us about how CLAHRCs work and are organised; how they have assessed any emergent impacts of CLAHRCs; and what strengths and limitations are apparent in the ways by which CLAHRCs have been evaluated to date.

## Methods

The protocol was registered in PROSPERO (Registration number CRD42016042945).

### Data sources and searches

We searched the following databases: CINAHL, MEDLINE, EMBASE, PsycINFO, Cochrane Methodology Register, Cochrane Central Register of Controlled Trials, Cochrane Database of Systematic Reviews, Database of Abstracts of Reviews of Effects, Health Technology Assessment, NHS Economic Evaluation Database, HMIC Health Management Information Consortium, SPORTDiscus, Scopus, TRiP database and PROSPERO. All the searches were restricted to English language only and were conducted in June 2016. Update searches were conducted up to June 2018 using the same search terms and databases. Details of the search strategies are available in Additional file 1.

As our focus was on identifying evaluations of CLAHRCs, we also searched for eligible studies in current and archived CLAHRC websites since we were aware that some CLAHRCs had carried out internal evaluations. Reference lists of retrieved articles were scanned to identify any additional studies.

### Study selection

Any published empirical papers drawing on data from an evaluation of CLAHRCs or some aspect of them were eligible for inclusion if they fulfilled at least one of the following criteria:

- an external or internal evaluation of the CLAHRC(s) or CLAHRC process,
- an exploration of the CLAHRC(s) as a novel organisational form and
- development of theory using the CLAHRC(s) as a research setting, i.e. including empirical data.

As our focus was on identifying evaluations of CLAHRCs as an entity, any evaluations that were based around a single project conducted within a CLAHRC were excluded from the review. This included descriptive accounts aiming to showcase the achievements of an individual project without providing rigorous evidence and/or critical analysis of these achievements; and/or (2) theory-building accounts that use a single project as an empirical illustration of a broader theoretical issue.

References were loaded onto the systematic review web app Rayyan QCRI [11] for title and abstract screening. Study selection was performed independently by one researcher and checked by a second. All full text studies that were provisionally excluded were discussed collectively by the research team.

### Data extraction and quality assessment

From the primary output paper for each identified evaluation, details of the type and main findings were extracted and assessed by one researcher and checked by a second. As NIHR funded studies are extensively peer reviewed and quality assured prior to publication, we did not undertake separate quality assessments for all four NIHR funded evaluations. The other included CLAHRC evaluations are presented descriptively with any major limitations in reporting highlighted.

### Method of synthesis

As the NIHR funded evaluations were mixed methods, and the other included evaluations were largely qualitative, we performed a narrative synthesis of the evidence. Consistent with an integrative approach to synthesising evidence, the narrative synthesis aimed to present a descriptive summary of findings across studies and then to generate, across reported findings, a number of themes relevant to the aims of this review. The original commissioning brief anticipated that evaluations may address organisational form, structure and processes, funding arrangements, nature of formative partnerships, engagement of health care users and the general public, emerging impacts and potential for sustainability of change [10]. We used these themes as a guiding framework to help answer our research questions on organisation, impact and evaluation. An iterative process of adaptation and refinement was undertaken by two researchers to generate initial themes, and these were further refined via consensus discussions with the full research team.

Given the interdisciplinary nature of CLAHRC work and the resulting diversity of papers being reviewed, it was particularly important to minimise individual disciplinary biases when synthesising the literature. This was accomplished through regular reflective discussions within the research team (which included two organisation and management scholars and two health services researchers) as well as through internal review from academic colleagues and CLAHRC managers in the role of ‘critical friends’.

## Results

**Fig. 1:**
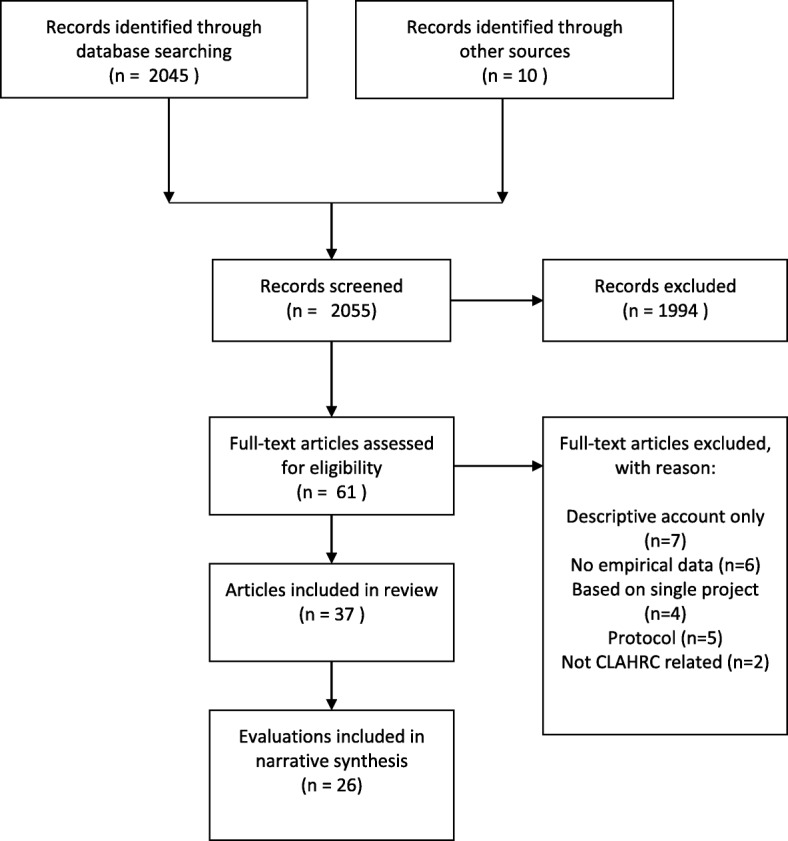
Flow Diagram of CLAHRC evaluations

After de-duplication, we identified a total of 2045 records through database searching and a further 10 records through other web based sources. Titles and abstracts were screened, and 61 full text papers were assessed for inclusion (see Fig. 1: PRISMA flow diagram).

### Excluded studies

We excluded 24 papers on eligibility grounds. Given our stated focus on emergent impacts, we did not include papers that presented descriptive accounts of CLAHRC(s) processes (*n* = 7) or those that were conceptual and not based on empirical data (*n* = 6). We also excluded studies that were based around a single project conducted within a CLAHRC (*n* = 4) rather than addressing a CLAHRC as a whole. Five protocols and two papers unrelated to the evaluation of CLAHRCs were also excluded. We checked the reference lists of excluded papers to ensure we had identified and included all relevant evaluations.

### Included studies

In total, 26 studies (reported in 37 papers) were deemed eligible for inclusion. We included all four NIHR funded independent evaluations of CLAHRCs [12–15] (also reported in a further 11 papers [16–26]). Details of the included evaluations and their associated outputs are presented in Table 1. The four NIHR funded evaluations were all longitudinal and mixed-methods by design and drew their conclusions from the analysis of more than one CLAHRC. Table 2 summarises the main findings from the each of the four NIHR funded evaluations. A further 22 studies [27–48] of aspects and processes of individual CLAHRCs were also identified, and these are presented in Table 3.

**Table 1:** NIHR funded evaluations of CLAHRCs

Title, years and funding	Design	Final report and related outputs
HS&DR-09/1809/1073: A formative evaluation of Collaboration for Leadership in Applied Health Research and Care (CLAHRC): institutional entrepreneurship for service innovation [12] 2009–2012 £550,000 PI: Prof Andy Lockett	Longitudinal mixed methods. Qualitative case studies combined interview data (174 in total across all nine CLAHRCs and 4 in-depth sites), archival data and observations, over a 4-year period. Quantitative social network analysis (SNA) to capture key actors individual networks of interaction across two points in time (2011, *n* = 81; 2013, *n* = 86).	https://www.journalslibrary.nihr.ac.uk/hsdr/hsdr02310/#/abstract Currie et al. (2013) [17] Currie et al. (2014) [16] Oborn et al. (2013) [18] Racko (2018) [19]
HS&DR-09/1809/1075: Networked innovation in the health sector: comparative qualitative study of the role of Collaborations for Leadership in Applied Health Research and Care in translating research into practice [14] 2010–2013 £575,000 PI: Prof Harry Scarborough	Mixed-methods in 2 temporal phases. Qualitative in-depth case studies with 3 CLAHRCs and 3 similarly networked innovation initiatives in the USA and Canada. Semi-structured interviews with key participants across CLAHRC cases (phase 1, *n* = 67; phase 2, *n* = 42) and North American cases (phase 1, *n* = 67; phase 2, *n* = 42) Social network analysis via the use of survey instruments across two time points with CLAHRCs (phase 1, *n* = 261/367; phase 2, *n* = 211/333) and one time point with one North American case (*n* = 39/77) Analysis of cognitions via the use of a cognitive mapping tool	https://www.journalslibrary.nihr.ac.uk/hsdr/hsdr02130/#/abstract D’Andreta et al. (2013) [21] D’Andreta et al. (2016) [20] Evans and Scarbrough (2014) [22]
HS&DR-09/1809/1072: Collective action for knowledge mobilisation: a realist evaluation of the Collaborations for Leadership in Applied Health Research and Care [13] 2010–2014 £600,000 PI: Prof Jo Rycroft-Malone	Longitudinal realist evaluation involving hypothesis generation, refining, testing and programme theory specification. Data derived from in-depth case studies of 3 CLAHRCs over four phases of data collection. Involved semi-interviews (*n* = 114 in total across all 4 phases), observation of one board meeting and two feedback sessions, documentary analysis and an interpretive forum involving members of seven CLAHRCs and other stakeholders (*n* = 28).	https://www.journalslibrary.nihr.ac.uk/hsdr/hsdr03440/#/abstract Protocol published 2011 [60] Rycroft-Malone et al. (2013) [24] Rycroft-Malone et al. (2016) [23]
HS&DR-09/1809/1074: Delivering the aims of the Collaborations for Leadership in Applied Health Research and Care: understanding their strategies and contributions [15] 2009–2012 £465,000 PI: Dr. Ellen Nolte	Mixed methods in two temporal phases. Phase 1 mapping involved documentary analysis in combination with interviews with senior individuals, workshops, mini-conferences and/or non-participant observation of key meetings (*n* = 48 participants across all nine CLAHRCs). Phase 2 explored three research questions identified in phase 1 through a stakeholder survey of six CLAHRCs (*n* = 242), in-depth case studies of two CLAHRCs (*n* = 29), validation interviews with all nine CLAHRCs and the funder (*n* = 18), and document review.	https://www.journalslibrary.nihr.ac.uk/hsdr/hsdr03250/#/abstract Ling et al. (2011) [25] Soper et al. (2013) [26]

**Table 2:** Main findings from the NIHR funded evaluations of CLAHRCs

Author, year	
Lockett, 2014 [12]	Local context and key service and research actors played an important role in shaping the initial design of the CLAHRCs. This initial design then ‘locked-in’ CLAHRCs to specific paths of development. Five different archetype models of CLAHRCs were identified: 1. Purposeful integration of multiple stakeholder groups to enable a multidisciplinary research process 2. Loosely autonomous research streams with designated knowledge brokers 3. Modular independence of research and implementation processes, separated to run in parallel 4. Collaborating through loose networks building on existing relationships which form the basis for collaboration 5. Centralised control over both research and knowledge translation (KT) activities through on-going accountability mechanisms and monitoring of project teams Two main forms of engagement were identified: work undertaken in signing up the CLAHRC stakeholders, and wnining over the hearts and minds of actors, which occurred through alignment activities and consensus building. Ability to do this was shaped by the nature of CLAHRC structures and also the professional status and role of actors. Four main forms of activity to embed CLAHRC were identified: (i) education, (ii) the creation of new roles, (iii) the embedding of tools and routines in practice and, finally, (iv) the construction of a CLAHRC identity. Across the CLAHRCs, there were differences in the manner in which CLAHRC focal actors sought to embed the CLAHRCs. The authors also found a significant degree of similarity across CLAHRCs over time, whereby CLAHRCs sought to learn lessons from other CLAHRCs. There were systematic variations in CLAHRC actors’ ability to bridge the research–practice boundary. But the CLAHRC initiative has led to the development of more relationships that span the research—practice divide.
Scarborough, 2014 [14]	Mechanisms of KT developed by the each CLAHRC were influenced by the vision and beliefs of their senior leadership teams and shaped by the emergent management practices. This in turn shaped the kinds of social networks that they developed and influenced the way different groups worked together. Analysis comparing CLAHRCs with each other, and with similar organisations in Canada and the USA, showed the impact of these differences in approach on each initiative’s ability to meet the challenge of getting research into practice. Where a CLAHRC framed KT as essentially involving the dissemination of high-quality evidence into practice, ‘bridging mechanisms’ of KT were utilised to overcome the boundaries between research and practice. Where a CLAHRC placed greater emphasis on the integration of research practices with practical concerns, ‘blurring’ of boundaries occurred to a much greater extent. There are different ways of doing this, and not a one-size-fits-all approach. Analysis of CLAHRC social networks highlighted the importance of both ‘closure’ (dense social ties within particular areas) and ‘brokerage’ (bridging ties across different groups) for a networked process of innovation. CLAHRCs were characterised as ‘ambidextrous’ network forms in that they need both ‘closure’ and ‘brokerage’ to support the process of innovation.
Rycroft-Malone, 2015 [13]	Opportunities for CLAHRCs to implement research in practice were influenced by the vision and views of those who set them up, including how they had structured the CLAHRCs. CLAHRC leaders played an important role in how the collaboration functioned. The academic-practice divide played out strongly as a context for motivation to engage, in that ‘what’s in it for me’ resulted in variable levels of engagement along a co-operation-collaboration continuum. More distributed leadership was associated with greater potential for engagement. Different positions and interpretations came together to result in a mixed picture of implementation. A number of approaches to mobilising knowledge were identified, including service improvement, making evidence accessible, mobilising local evidence, paying attention to aspects of implementation in the conduct of research, and using home-grown evidence. The balance of activity was weighted towards research production rather than its use in practice and towards knowledge transfer-type approaches rather than co-production. The creation of boundary spanning roles was the most visible investment in implementation, and credible individuals in these roles resulted in cross-boundary work, in facilitation and in direct impacts. There were examples of CLAHRC activity having an impact on the way that services were delivered to patients and in providing opportunities for practitioners and researchers to come together to share ideas and do joint projects. Learning within and across CLAHRCs was patchy depending on attention to evaluation.
Soper, 2015 [15]	CLAHRCs were rooted in local relationships, built around matched funding from NHS organisations, local capacity and expertise. The local remit supported the development of collaboration, encouraged responsiveness to local research needs and shaped the separate character of each CLAHRC. CLAHRCs demonstrated a clear drive to promote integration and used clinical and managerial knowledge brokers such as ‘locality leads’, ‘diffusion fellows’ or ‘CLAHRC Associates’ to encourage their peers to become involved in research. There was some evidence that academics were becoming more interested in needs-driven research and that commissioners were seeing the CLAHRCs as a useful source of support. There was growing recognition that sustaining collaboration across sectors as well as within sectors requires iterative and continual engagement between clinicians, academics, NHS commissioners, managers and patients. Despite initial challenges, the CLAHRCs succeeded in engaging different stakeholder groups although some CLAHRCs were less successful with some groups, such as mid-level NHS management, than others. Partnership working, responsiveness and the co-production of research were seen as core to promoting and sustaining engagement. Exposure to people from other disciplines and other backgrounds helped to broaden mutual understanding of implementation’ and of other research fields and methodologies. Over time, the NHS focus on producing change in (clinical) practice was seen to be just as important as the academic focus on producing good-quality research. Communication with commissioners was supported by the development of a CLAHRC ‘brand’, which helped to identify CLAHRC products and give them credence. The CLAHRCs were increasingly seen as useful sources of sound evidence to support (and prompt) constructive dialogue between commissioners and providers.

**Table 3:** Other evaluations of individual CLAHRCs

Author, year and aims	Design	Main findings
Ariss, 2012 [27] Early internal evaluation of CLAHRC South Yorkshire (only executive summary publicly available).	Described as a developmental evaluation approach combined with realist evaluation and a utilisation focus to guide the evaluation activities. Informed by 27 interviews with theme leads, programme managers, members of the core team and the executive board, conducted in 2011. Analysis of quarterly theme activity reports covering the period from October 2008 to March 2010 (53 in total).	Implications and opportunities are reported under nine interlinked headings: 1. Changing landscape 2.Participation, involvement and engagement 3.Public and patient involvement 4.Priority setting 5.Addressing inequalities 6.Capacity building 7.Governance and programme processes 8.Funding and value 9.Outcomes and impact On impact, the importance of leaving a ‘footprint’ or evidence of the legacy of CLAHRC SY is recognised. However, there is a danger that collaboration and other successes could remain invisible or unattributed and impacts occur further ‘downstream’ and beyond the CLAHRC funding envelope.
Caldwell, 2012 [28] How national-level understanding of the aims and objectives of the CLAHRCs translated into local practice in North West London.	Uses a variation of Goffman’s frame analysis to trace the development of the initial national CLAHRC policy to its implementation at three levels. Data collection and analysis were qualitative through interviews (*n* = 21), document analysis and observation (hours not specified). Interviews conducted at two different time periods but by different interviewers for slightly different purposes but are an acknowledged limitation.	Analysis at the macro (national policy), meso (national programme) and micro (North West London) levels showed a significant common understanding of the aims and objectives of the policy and programme. Local level implementation in North West London was also consistent with these.
Chew, 2013 [29] Explore the enactment of full-time intermediary roles in bridging the knowledge-translation gap.	A qualitative case study in an anonymised CLAHRC exploring the formalised intermediary roles of seven ‘knowledge brokers’ enacted in different partner organisations. Data collection included individual interviews, a focus group with all intermediaries and 118 h of workplace ethnographic observation.	Structural issues around professional boundaries, organisational norms and career pathways may make such roles difficult to sustain in the long term. Despite the intuitive appeal of intermediary roles as a knowledge-translation solution, organisations should think carefully about how best to realise them if they are to achieve their potential in a sustainable manner.
Cooke, 2015 [30] Explores how one CLAHRC used collaborative priority setting between researchers and end users to shape its research agenda and project development.	Mixed methods Semi-structured interviews (*n* = 28) with CLAHRC researchers and partners. Field notes from a workshop of key stakeholders. Documentary analysis of CLAHRC internal reports and annual reports from the first two and half years (2008–10).	Dedicated CLAHRC resources, including the use of ‘matched funding’, increased the potential for engagement across academic and practice boundaries.
Fitzgerald, 2015 [31] Explores how the design and initiation of a CLAHRC impacts its modes of operation and knowledge mobilisation.	Longitudinal case study of one CLAHRC in first 3 years of existence (2008–2011). Documentary analysis of minutes from board and project team meetings combined with observation and participation in project meetings of two implementation project teams.	Setting up translational networks is insufficient in itself. To leverage benefit attention must be paid to devising a structure which integrates research production and use and facilitates lateral cross-disciplinary and cross-organisational communication Knowledge mobilisation extends beyond knowledge translation to include the negotiated utilisation of knowledge—a balanced power form of collaboration.
Gerrish, 2014 [32] Evaluation of an initiative undertaken by NIHR CLAHRC South Yorkshire to increase KT capacity among clinical and academic nurses from partner organisations through a secondment model.	Qualitative evaluation using focus group and individual interviews with 14 clinical and academic secondees and five managers from partner organisations to explore contribution secondees made to KT projects. Qualitative content analysis used to identify criteria for success.	Six criteria for judging the success of the secondments at individual, team and organisation level were identified: KT skills development, effective workload management, team working, achieving KT objectives, enhanced care delivery and enhanced education delivery. Hosting teams should provide mentorship support to secondees and be flexible to accommodate secondees’ needs as team members. Ongoing support of managers from seconding organisations is needed to maximise the benefits to individual secondees and the organisation.
Heaton, 2015, 2016 [33, 34] To delineate the mechanisms by which, and circumstances in which, some projects carried out under the programme achieved success in knowledge translation while others were frustrated.	Longitudinal case study of one CLAHRC over 5 years of existence (2008–12). Phase 1 mapping to develop programme theories involving 77 semi-structured interviews with 54 stakeholders, combined with documentary analysis. Phase 2 exploration of programme theories via in-depth case studies of four CLAHRC projects. Twenty-eight semi-structured interviews with project teams.	Identification of five rules based on nine associated mechanisms for promoting knowledge translation through collaborations based on principles of co-production (active agents, equality of partners, reciprocity and mutuality, transformative and facilitated). 1. Base research on co-production through closer collaboration 2. Establish small strategic teams led by strong facilitative leaders 3. Harness and develop respective assets 4. Promote relational adaptive capacity 5. End user is king
Howe, 2013 [35] To identify whether an assessment framework can provide information for collaborative leaders about how the collaborative approach is implemented, what and where additional support may be required and identify potential peer exemplars.	Assessment of extent to which 17 projects engaged with eight promoted collaborative methods (2010–2011). Review of 17 individual formal ‘end of project’ meetings. Two to five participants in each meeting and included use of two non-linear scales (0–6) to assess the relative priority given to ‘engagement’ and ‘results’.	Uptake of collaborative methods was variable across projects with no project engaging with all methods, but all engaging with some.
Jordan, 2014 [36] Explores the nature of the research team–service user relationship in collaborative health research conducted by CLAHRC for Nottinghamshire, Derbyshire, and Lincolnshire (NDL).	Utilises data from a proposed internal evaluation of the CLAHRC-NDL [61] (no data presented) focused on the CLAHRC as a developing organisation and exploring members’ experiences of the CLAHRC. The study ‘involves’ 46 semi-structured interviews from across one CLAHRC’s membership. The authors state that they intentionally prioritise the service user voice but unclear how many (if any) services users are included in the dataset.	There can be a disparity between initial expectations and actual experiences of involvement for service users. Therefore, as structured via ‘The Three Rs’ (Roles, Relations and Responsibilities), aspects of the relationship are evaluated (e.g. motivation, altruism, satisfaction, transparency, scope, feedback, communication, time). Regarding the inclusion of service users in health research teams, a careful consideration of ‘The Three Rs’ is required to ensure expectations match experiences.
Kislov, 2012 [37] Explored intra- and inter-organisational boundaries on the implementation of service improvement within and across primary healthcare settings and on the development of multi-professional and multi-organisational communities of practice in the Chronic Kidney Disease theme of the CLAHRC Greater Manchester (GM).	Qualitative embedded case study design, encompassing 20 semi structured interviews with practice doctors, nurses, managers and members of the CLAHRC facilitation team. Data also derived from 20 h of direct observation, conducted predominantly at learning sessions and practice meetings and from documentary analysis.	The study showed that in spite of epistemic and status differences, professional boundaries between general practitioners, practice nurses and practice managers co-located in the same practice over a relatively long period of time could be successfully bridged, leading to the formation of multiprofessional communities of practice. While knowledge circulated relatively easily within these communities of practice, barriers to knowledge sharing emerged at the boundary separating them from other groups existing in the same primary care setting. The strongest boundaries lay between individual general practices, with inter-organisational knowledge sharing and collaboration between them remaining unequally developed across different areas due to historical factors, competition and strong organisational identification.
Kislov, 2014 [38] Exploration of the discontinuity of knowledge sharing across different groups co-located within the collaborative research partnership, CLAHRC GM.	Qualitative single case study involving a purposive sample of 45 research participants drawn from both core and peripheral membership of the four domains of CLAHRC GM. Interviews were supplemented by direct observation (69 h) of various boundary encounters (e.g. implementation team meetings, learning sessions, practice visits, etc.). Document analysis of (e.g. reports, meeting minutes, presentations, leaflets, etc.) was also carried out.	The structure of the CLAHRC institutionalised the pre-existing gap between the activities of research and implementation strands underpinned by political (conflicting goals and incentives) and epistemic (conflicting attitudes to evidence) factors. This prevented an open conflict between the strands, but at the same time removed the need to renegotiate the boundary and develop a shared practice. Collaboration within the CLAHRC is a complex, dynamic system of practices, boundaries, and boundary bridges with the potential for both continuity and discontinuity in knowledge sharing. Differences between communities of practice give rise to discontinuities in knowledge sharing. This in turn highlights the role of fragmented organisational structure, divergent meanings and identities, and marginalised boundary bridges in the (re)production, legitimisation, and protection of boundaries.
Kislov, 2016 [39] Explored what strategies knowledge brokering professionals deploy to alleviate the challenges associated with fulfilling a hybrid role in CLAHRC GM and examines the implications of these strategies for theoretical understanding of knowledge brokering in a broader organisational and institutional context.	Qualitative embedded case study design involving 57 research participants drawn from three projects and the management team to represent different sectors (primary, community and secondary care) and occupational groups (doctors, nurses, care coordinators, managers, etc.5). Semi-structured interviews were conducted at two points and were supplemented by direct observation (14 h) of team meetings, educational sessions and practice visits, as well as by numerous informal face-to-face conversations with participants.	Formally designated knowledge brokers mitigate the constraining power of context by transferring some of their knowledge brokering functions to managers and clinicians; by conforming to the local ways of doing things; and by complementing (and even replacing) the situated processes of knowledge brokering with the supply of knowledge and skills to clinicians wishing to achieve their organisational performance objectives. These strategies reveal how, through use of knowledge brokers, macro-level institutional arrangements exert influence on the dynamics of knowledge processes unfolding in practice, how the formalised and emergent elements of knowledge brokering as a collectively enacted phenomenon are intertwined, and how the professional expertise and authority of hybrids can become an impediment to their knowledge brokering function. Initiatives deploying designated boundary spanning roles could possibly benefit from diversifying the pool of knowledge brokers to include managers, quasi-managerial professionals and professionals with formal managerial responsibilities, and supporting the formation of links between knowledge brokers working at different levels.
Kislov, 2017 [40] Explored how investment in boundary spanning roles, processes and practices changed over time in CLAHRC GM.	Qualitative longitudinal case study involving 88 participants. Semi-structured interviews (30 to 95 min in duration) were conducted (in two rounds (2009–2010 and 2012–2013). CLAHRC facilitators and managers remained in their posts for the second round of data collection and were interviewed twice. However, as different general practices participated in CLAHRC projects in 2009–2010 and 2012–2013, the sample significantly differed between the two rounds, but remained comparable in terms of the professional and organisational groups represented. A focus group was conducted with all facilitators at the end of 2013 to discuss the development of legitimacy over time.	Using a Bourdieusian lens, three main themes emerged: (1) changes in the distribution of economic, cultural and social capital mobilised by boundary spanners; (2) implications of these changes for the relationships between the intersecting fields; and (3) effects on the social trajectories of boundary spanners. The legitimation of boundary spanning roles and practices is a highly transformative, collective and political process that increases the capital endowments and authority of individual boundary spanning agents but may lead to the erosion of the very same roles and practices that were being legitimised
Marston, 2013 [41] Investigated how PPI was put into practice and how patient and professional roles developed over time.	A 4-year ethnographic study, using participant observation of PPI activities run by CLAHRC Northwest London (NWL) (160 h) and in-depth interviews (*n* = 89), 45 with patient participants (i.e., patients and service users involved in CLAHRC improvement projects) and 44 with health-care professionals involved in implementing PPI.	At first, health professionals demanded evidence of PPI effects of the type typical in clinical practice, such as cost-effectiveness data, treating PPI as a discrete intervention to improve a specific health outcome. They often spoke about effect in linear terms, and measured success using indicators such as successful participant recruitment and retention or tangible non-health outputs (e.g. leaflets co-designed with patients), rather than changes in health outcomes. Patients talked about their own contributions in collective and utilitarian terms: they were reluctant to attribute success to individuals, emphasising the role of the team. For them, effect meant timely (and rapid) implementation of incremental changes in health care, which were then sustained and improved upon through collaborative relationships between patients, clinicians, researchers, and others. Over time staff focus shifted towards creating environments conducive to patient collaboration, and less on calculating the effect of individual contributions. PPI success increasingly described in terms of collaborative relationships between diverse patients and professionals, and acknowledged the importance of unpredictable positive effects of patient innovations.
Martin, 2013 [42] Explored the way in which CLAHRC for Leicester, Northamptonshire and Rutland (LNR) was put into practice, to understand the theories of change on which its structures and activities are premised, and the degree to which these are realised.	Longitudinal, mixed-methods using interviews, observations and documentary analysis. Twenty-seven interviews (conducted 2010–2011) with core CLAHRC staff including the executive group, theme leads, deputy leads and managers responsible for the programme of research and implementation. Supplemented by ‘extensive’ observational work involving attendance at key CLAHRC internal meetings and externally-oriented events. Documentary analysis of minutes of meetings, strategy documents and externally oriented publicity materials were used not as a data source but rather to sensitise the researchers to key issues in the CLAHRC and inform interviews.	The breadth of CLAHRCs’ missions seems crucial to mobilise the diverse stakeholders needed to succeed, but also produces disagreement about what the prime goal of the CLAHRC should be. A process of consensus building is necessary to instil a common vision among CLAHRC members, but deep-seated institutional divisions continue to orient them in divergent directions, which may need to be overcome through other means.
Reed, 2018 [44] Consolidation of cross-project learning from the first 5 years of the CLAHRC NWL (2008–2013).	Learning from 22 evidence translation projects to used develop theory and a conceptual framework. Authors acted as auto-ethnographers drawing on own experiences in running and researching the CLAHRC NWL programme. Analytical auto-ethnography combined with documentary analysis (including project proposals, progress and final project reports, posters and presentations) and a non-systematic literature review. Results were interpreted using complexity theory and ‘simple rules’ that enhanced project progress were identified.	Three strategic principles, (1) ‘act scientifically and pragmatically’—knowledge of existing evidence is only one part of the effort required to achieve sustainable improvements in care in complex systems; (2) ‘embrace complexity’—evidence-based interventions only work if supporting or dependent practices and processes of care are working sufficiently well; (3) ‘engage and empower’—evidence translation and system navigation requires commitment and insights from staff and patients with experience of the local system, and changes need to align with their motivations and concerns. Twelve associated ‘simple rules’ also presented to provide actionable guidance to support evidence translation and improvement in complex systems.
Renedo, 2015 [43] Examined how PPI was organised and enacted in practice in CLAHRC NWL.	Uses data drawn from the larger ethnographic study by Marston [41]. Draws on interviews with 20 ‘patient participants’—patients or carers involved in CLAHRC improvement projects conducted between September 2010 and November 2012 and supplemented with 132 h of observation of PPI activities run by the CLAHRC.	Patients used four elements of organisational culture as resources to help them collaborate with healthcare professionals. The four elements were (1) organisational emphasis on non-hierarchical, multidisciplinary collaboration; (2) organisational staff ability to model desired behaviours of recognition and respect; (3) commitment to rapid action, including quick translation of research into practice; and (4) the constant data collection and reflection process facilitated by improvement methods.
Spyridonidis, 2015 [46] To explore the relationship between knowledge translation and leadership in a CLAHRC aiming to bridge the gap between research and practice.	Undertaken as part of a longitudinal 5-year exploration of the development of a CLAHRC. Data for study derived from interviews with the clinical leaders (*n* = 36) of CLAHRC projects at two time points. Supplemented with documentary analysis of internal CLAHRC reports	Relationship between leadership and KT shifted over time from an authoritarian top-down leadership with set outcome measures for KT performance to one of distributed leadership that better accommodated the diverse range of CLAHRC stakeholders. Knowledge translation viewed as an on-going process informed by interactions between individuals and groups, underpinned by pre-existing individual and group experiences and values.
Spyridonidis, 2015 [45] Explored the organisational development of the CLAHRC with a focus on a new hybrid physician–manager role, working within this new organisational form.	Longitudinal qualitative study in CLAHRC NWL. Data derived from interviews with physicians who had taken on a hybrid physician–manager role (*n* = 62) in CLAHRC projects. Physicians were interviewed twice, at the beginning of their project and at the end over an 18-month time period (total *n* = 124 interviews). The study also draws on interviews with CLAHRC senior members conducted as part of the larger study.	Three differing responses were found to taking on a hybrid physician–manager role (the sceptics, the innovators and the late majority), with identity emerging as a mitigating factor for negotiating potentially conflicting roles.
Waterman, 2015 [47] To explore how CLAHRC GM knowledge transfer associates facilitated the implementation of evidence-based healthcare across several commissioning and provider health care agencies.	A prospective co-operative inquiry with eight knowledge transfer associates responsible for the facilitating the implementation of evidence based practices in six projects in primary- and community-care. Twenty semi-structured interviews with other team members to gain their perspectives of the facilitation role and process.	Facilitation is context dependent and ‘one size does not fits all’. Facilitators need tailored support and education to enhance their capacity to support the process of implementation.
Wright, 2013 [48] Sought to understand the experiences of nurses and allied health professionals acting as first-time knowledge brokers and those of their mentors in CLAHRC NDL.	Exploratory study using interviews with 17 knowledge brokers and 5 mentors to elicit their experiences as first-time knowledge brokers, attempting to bridge the research-practice gap within CLAHRC NDL. Supplemented with data from documents (reflective diaries and final reports) produced as part of the programme.	Four themes described their experiences: expectations, pragmatics, emotional reactions and outcomes. Knowledge brokering roles had multi-level benefits. However, there is a lack of support and recognition for these roles at an organisational level, making these activities difficult to sustain in the long term.

#### Synthesis of findings

Five prominent themes were identified from the literature: organisational form and emergent properties, the nature and role of boundaries, the deployment of knowledge brokers and other hybrid roles to support knowledge mobilisation, engagement of health care users and the general public in the form of patient and public involvement (PPI), and capacity building. We describe each of these themes in turn.

#### Organisational form and emergent properties

All the NIHR funded evaluations highlight the influence of local context and the interplay between local research producers and the key health service actors in shaping the initial design and organisational form of each CLAHRC. Drawing on a comparison of all nine CLAHRCs, five different knowledge translation ‘archetypes’ have been proposed to represent the different ways of achieving the balance between research production and research implementation (see Lockett et al., Table 2) [12]. However, Fitzgerald and Harvey caution that the rigid structural design of a CLAHRC may adversely impact its performance, particularly if the adopted form does not readily facilitate the intended function of knowledge mobilisation [31].

According to Soper et al., [15] key features of the CLAHRCs include a range of knowledge mobilisation approaches, efforts to promote cultural change and freedom to experiment, learn and adapt while Rycroft-Malone et al. [13] identified collaborative action, relationship building, engagement, motivation, knowledge exchange and learning as key mechanisms important to the processes and outcomes of CLAHRCs.

The way each CLAHRC developed was highly influenced by the vision and beliefs of their leaders; they shaped the type of resulting social networks, and the way different groups worked together [14]. Senior leaders and managers played an important formative role in selecting, enacting and interpreting different knowledge mobilisation practices [21]. Many were well-known clinical academics and relied on existing relationships to support early mobilisation activity. But in doing so, they may also have restricted the development of novel, integrated approaches to the production and implementation of applied health research [17]. Despite this, it was shown that the CLAHRC initiative led to the development of relationships that span the ‘research to practice’ divide and have been able to work across professional and organisational boundaries [12].

#### Nature and role of boundaries

Multiple types of boundaries were highlighted across evaluations. Rycroft-Malone et al. [13] suggest that the way in which CLAHRCs had developed their organisational form resulted in the reinforcement, rather than resolution, of boundaries between research and practice, between higher education and health services and between communities. They argue that the different perspectives which individuals and groups brought to the issue were a function of, and perpetuated, professional and epistemic boundaries [49]. The geographic delineation of the CLAHRCs resulted, in turn, in physical and spatial boundaries. Similarly, Kislov describes the boundary between the research and implementation activities that gives rise to discontinuities in knowledge sharing within one CLAHRC, [38] whereas Currie et al. [16] describe epistemic differences and power struggles unfolding between health services researchers and organisation scientists in relation to the CLAHRC activities.

Analysis by Scarbrough et al. [14] focused on the differences between ‘bridging’ and ‘blurring’ approaches to boundary spanning. Where a CLAHRC framed knowledge mobilisation as the dissemination of high-quality evidence into practice, ‘bridging mechanisms’ were utilised to overcome the boundaries between research and practice. In contrast, where greater emphasis was placed on the integration of research practices with practical concerns, ‘blurring’ of boundaries occurred to a much greater extent. Scarbrough et al. [14] argue that reliance on these different mechanisms seems to reflect the relative extent of ‘epistemic’ differences between the communities involved as well as the specific local configurations of contextual factors. Furthermore, they suggest both approaches could be used simultaneously as what determines their appropriateness is ‘not the model per se, but rather the interplay between an initiative’s specific context and unfolding role-enactment and work-practices’ [22].

Whilst the evaluative literature focused mainly on developing the theory around the concepts of boundaries and boundary spanning, some useful practical implications were also drawn. CLAHRCs should ‘diagnose’ the existing professional and organisational context when implementing knowledge mobilisation projects, [37] actively facilitate the negotiation of concepts, approaches, and objectives that are interpreted in conflicting ways by different groups, create incentives to support productive joint working, and articulate the overarching goals and philosophy of a collaborative enterprise at early stages [38]. Drawing on the internal evaluation of one CLAHRC, Martin and colleagues demonstrate that deep-seated institutional divisions between CLAHRC members were ‘overcome’ by concerted action resulting from the External Advisory Review [42].

#### Deployment of knowledge brokers and other hybrid roles

A number of evaluations explored the use of knowledge brokering and ‘hybrid’ roles to support knowledge mobilisation within the CLAHRCs. These types of roles are often proposed as a means to overcome ‘boundaries’. Although often seen as a promising solution to the problem of bridging the second translational gap, evaluations highlight that there is often lack of support and recognition for these roles at an organisational level, and that formidable professional boundaries, existing organisational norms and lack of institutionalised career pathways for knowledge brokers may make such roles difficult to sustain in the longer term [29, 48]. The potential of formalised knowledge brokering roles can also be decreased by over-formalisation, infrequency of interaction, competition for recognition and resources, low trust and lack of rewards [38]. Scarbrough et al. [14] also show that in more decentralised structures, lack of clarity of the nature of the role specifications may limit the effectiveness of knowledge brokering.

In their study of clinicians seconded to roles as formalised knowledge brokers, Kislov et al. [39] describe the strategies such clinicians deploy to surmount challenges associated with bridging multiple boundaries: (1) relying on additional boundary ‘bridges’, (2) conforming to existing ways of doing things and (3) shifting from ‘facilitating’ to ‘doing’. Their analysis sheds new light on the limitations of clinicians as designated knowledge brokers, demonstrating that, paradoxically, professional authority can sometimes become an impediment to the successful realisation of all dimensions of knowledge brokering.

In a broader study into the evolution of formalised knowledge brokering roles over time, Kislov et al. [40] discuss how knowledge brokers accumulate, convert and mobilise different forms of ‘capital’ to achieve legitimacy with multiple stakeholder groups. Unintended (and largely unexpected) consequences of legitimation include exclusion of some stakeholder groups (for example, academic researchers) from bridging the gap between research and practice as well as the gradual transformation of ‘knowledge brokers’ into ‘managers’, with a corresponding decrease in their brokering activities on the ground.

Finally, at an individual level of analysis, Spyridonidis et al. [45] describe that by creating hybrid physician-manager roles that make sense to professionals, so as to enable knowledge mobilisation, some (the ‘innovators’) easily nested this role within their existing professional identity. Others (the ‘sceptics’) found it much harder and believed that it might erode their professional autonomy. Many who initially resisted (the ‘late majority’) eventually came around, once they could redefine the role as one more around clinical leadership.

#### Engagement of health care users and the general public

None of the NIHR funded evaluations had a particular focus on PPI; other evaluations of relevant structures and processes were also relatively scarce. Soper et al. [15] did interview PPI representatives from two CLAHRCs as part of their case studies. They suggest that where the CLAHRC had existing expertise and relations they could, and did, build strong relations with such stakeholders.

Three studies investigated how PPI was enacted and how patient and professional roles developed over time in CLAHRCs [27, 41, 43]. One of these describe how patients were able to draw on elements of organisational culture (such as an emphasis on non-hierarchical, multidisciplinary collaboration) to help them collaborate with healthcare professionals, [43] whilst another explores how patients’ views on PPI differ from those of healthcare professionals [41]. This latter study highlights the need to not only take patient voices into account but also to track the dynamic social processes and networks through which PPI can make a contribution to health-care improvement efforts. Given the ostensible requirement for collaborative partnership with patients, it is likely that authentic efforts to achieve this in practice will result in the same complexities being encountered as covered in themes above, requiring the same attention and consideration to navigate.

#### Capacity building

Increasing the capacity to undertake and use applied health research in the NHS and to foster a culture of collaboration between the academic and service delivery sectors was one of the key objectives that CLAHRCs were required by NIHR to address. Soper et al. surveyed NHS and academic staff across six CLAHRCs and found that both NHS and academic respondents strongly supported both of these aims. Although these aims were well understood, there was considerable uncertainty about how best to achieve them in practice, and CLAHRCs themselves felt that 5 years was too short a time in which to embed their approach and change the ‘norms’ of the service [15].

A small individual evaluation exploring capacity building in one CLAHRC suggests criteria for judging the success of capacity building secondment arrangements [32]. The study describes an experiential model of capacity development and reports different experiences of academic and clinical secondees. The academic secondees reported considerable personal development, but there was no evidence that secondments led to further involvement in research. Clinical secondees benefited from ongoing clinical engagement helping to maintain their credibility with staff whose practice they sought to influence. Findings also suggest that secondees required mentorship from host teams and support from managers in seconding organisations to maximise the benefits to individual secondees and to the organisations involved.

## Discussion

A significant investment was made in independent external evaluations of the ‘pilot’ CLAHRC initiative by NIHR. In addition, others (mainly funded through individual CLAHRCs) also carried out and published evaluations. To our knowledge, this review represents the first attempt to systematically capture learning from these sources. Evaluations have largely focused on describing and exploring the leadership, vision, values, structures and processes of CLAHRCs, the nature and role of boundary spanning and hybrid roles, the deployment of knowledge brokers and other hybrid roles to support knowledge mobilisation.

The relative lack of data about the early impact of CLAHRCs on health care provision or outcomes, whilst understandable due to the inevitable time lag between an intervention and its impact, is notable. To date, no systematic assessment of impact appears to have been made nor do there appear to be any plans in place to assess this. Assessing outcomes and sustainability requires a sufficient timeframe, and it would be difficult to expect that the NIHR funded evaluations could fully address these issues so early in the development of CLAHRCs. However, reflecting on the impact of the CLAHRCs was an original commissioning aim, and the opportunity to at least develop and share formative learning on the nature and type of impacts appears to have been missed. As no further funding for independent evaluations was made available by NIHR beyond that for the initial ‘pilot’ phase of CLAHRCs, longitudinal insights are also lacking.

This opportunity foregone may be a feature of the funded evaluations themselves and may reflect a preoccupation with the need on the part of the evaluators to generate high quality academic outputs over providing more pragmatic insights into what works, how and why. Indeed, we have found that much of the evaluative focus has led to the development of theory around emergent properties and processes. Whilst theory provides a foundation for further scientific insight, evidence on the impact of many of the emergent properties of CLAHRCs, particularly in relation to the knowledge mobilisation processes and roles that were adopted remains sparse. There is a large body of practical experience and learning that CLAHRCs will have gained from their work. However, much of this learning is currently ‘locked up’ [52] with the CLAHRCs themselves, undermining the further development of international knowledge base on research use and impact. Indeed, Davies et al. [52] highlight that these new models of partnership, which have aimed to improve the research to practice gap, have instead perpetuated a gap in our understanding of the effects of knowledge mobilisation in practice.

The role of capacity building as both a contributing process and an intended outcome in itself needs to be further examined. Given that capacity building was one of the three main objectives of the CLAHRCs [50, 51], relative scarcity of empirical data on how it was (or was not) enacted in practice, highlights a significant area for future research. In light of Gerrish et al. [32] reporting different experiences of academic and clinical secondees, it is necessary to better differentiate pathways to capacity building depending on the target group(s) involved (e.g. academics, clinicians, managers or hybrid roles) as well as recognise that enhanced capacity should be considered not only at individual level, but also at the level of teams and organisations. In particular, it is important to understand not only the impacts on capacity of the partner organisations, (the focus of [Soper’s evaluation [15]), but also develop and test ways of developing the capacity of academics themselves to deliver co-production projects. The latter should take into account both capacity to produce impact through knowledge mobilisation and capacity to produce high-quality research despite conflicting priorities and workload pressures.

We recognise that the range of knowledge mobilisation approaches adopted by CLAHRCs reflects the different personal, professional and organisational contexts in which they have evolved. As such, knowledge mobilisation is inherently complex, and the mechanisms through which activities produce intended (or unintended) outcomes can be highly context-dependent, making any evaluation challenging. Given the problem of attribution and the time lag between the end of an intervention and its medium- and long-term outcomes, the preference for formative, as opposed to summative, evaluations in the extant literature is hardly surprising. In addition, the intermediary position of knowledge mobilisation at the conflict-laden interface of policymaking, management, science and professional practice is likely to further politicise any evaluation attempts and affect the utilisation of their outputs.

Multiple questions remain about the ways in which evaluations could inform the actual practices of knowledge mobilisation despite the political tensions described above. If further evaluation is to be helpful to those involved in current and future collaborative partnerships such as CLAHRCs, as well as those developing methods of collaboration and co-production between research users and producers more generally, there remains a need to move beyond ‘cataloguing’ [53] to testing and linking these adopted and adapted strategies to impacts and outcomes. This should include novel methodological work developing or critically analysing the use of quantitative, qualitative and mixed methods to deliver timely, relevant and rigorous summative evaluations of deliberate knowledge mobilisation strategies in a range of settings and contexts. However, who should do this remains unclear. Knowledge production and mobilisation are a key focus in many of the current CLAHRCs and, understandably, any further reflection, self-evaluation and/or critical examination of the process of research itself may not be seen as a major priority. Any locally funded evaluation is also likely to be under-resourced as a result.

The relative lack of data on the practical implications and evidence-based ‘lessons learnt’ (beyond those developed within one CLAHRC [44]) for those who are actually ‘doing’ CLAHRC business is notable, despite the particular emphasis on sharing formative learning with the CLAHRCs within the original commissioning brief [10]. The developing academic literature (where this might not be expected to constitute a key element) does not appear to be complemented by publicly accessible literature with a more pragmatic ‘how to do’ focus. The benefit to practice of the large funding invested in evaluations of the pilot CLAHRCs by NIHR is not evident from this analysis in terms of outputs or timing, especially given that the second round of CLAHRCs started in 2014, before any of the findings from the NIHR funded evaluations were published.

Some other topics have received relatively little evaluative attention: role CLAHRCs can or should play in supporting sustainability and scale-up, the nature and extent of collaboration between and across CLAHRCs, the effects of co-production on the nature, scope and quality of research conducted by CLAHRCs. The Directors of the early CLAHRCs also identified challenges from their perspective; these included maintaining and sustaining resources dependent on matched funding arrangements, ensuring that a full range of NHS professional groups are engaged, the need to demonstrate both academic outputs and improvements in care [54]. But these too appear not to have been given much attention in funded evaluations.

It is widely recognised that there is a need for greater evaluation of the outcomes of patient involvement, [55, 56] but this synthesis demonstrates that identifying the dynamic processes and networks through which PPI can make a contribution to health-care improvement efforts within organisations like CLAHRCs is also crucial. Given that health research in the UK operates with a more explicit distinction of the roles of ‘patients’ and ‘professionals’ (in contrast to, for example, integrated knowledge translation or community-based participatory research efforts in North America [57]), it is vital to understand how collaborative organisations such as CLAHRCs can effectively extend this collaboration to encompass service users as well as service providers.

This review is not without limitations. First, we have deliberately focused on the emergence of one specific type of large-scale knowledge mobilisation initiative, and we are conscious that the findings of this review are to a certain degree shaped by the UK context. At the same time, our findings are likely to be applicable to a range of knowledge mobilisation partnerships (and their evaluations) internationally as the institutional pressures are similar across high-income countries [51, 58]. Second, due to our focus on empirical papers that report evaluation findings, we have excluded a number of conceptual papers that have been directly informed by their authors’ experience of designing and or working within CLAHRCs. Finally, we have decided against making formal judgements about the methodological rigour of individual evaluations as criteria for assessing research quality vary broadly depending on the epistemological position of the assessor; [59] instead, this review has adopted a pragmatic, pluralistic and epistemologically tolerant approach.

There is still much to learn about how the processes adopted and adapted by each CLAHRC actually deliver impact. CLAHRCs (and indeed other similar research and practice partnerships internationally) remain a rich and fertile research setting for those interested in the mechanisms, practices and consequences of knowledge mobilisation approaches and in the effects of models of research and practice partnership more generally. However, if future evaluations are to be more useful, then they need to heed the lessons of the past and deliver learning on mobilisation processes and impacts in a timely manner that can inform and influence the on-going development of such partnerships, thus bridging the gap between implementation science and the practice of implementation. We summarise our recommendations for further evaluation of research and practice partnerships as follows:

1. Emphasis should be placed on comparative evaluations that are embedded across research and practice partnerships (nationally or internationally), facilitating greater contextual understanding of what works, where, how and why.
2. Evaluations should explicitly capture, analyse and report knowledge mobilisation strategies employed and their impacts.
3. Given the complex multi-stakeholder context of research and practice partnerships, evaluations should aim to report perspectives on impact from different partners.
4. Reporting of unintended outcomes as well as contextual and/or political factors affecting mechanisms of impact should be encouraged.
5. Capacity building and PPI should be evaluated and reported taking into account the diversity of audiences and patient populations involved.
6. Evaluation outputs should themselves be accessible to non-academic audiences and generate actionable insights to surface pragmatic and experiential knowledge.

## Conclusions

Much of the evaluative focus on CLAHRCs has been on how they have been organised and on the development of theory around emergent properties. Evidence is lacking, however, on the impact of CLAHRCs, particularly in relation to the knowledge mobilisation processes and practices adopted. Further evaluation focused on which knowledge mobilisation approaches work, where, how and why in research and practice partnerships is warranted.

## Additional file

Additional file 1: Search strategy for CLAHRC evaluations. (DOCX 15 kb)
