# Supplementary material for: Learning from the emergence of NIHR Collaborations for Leadership in Applied Health Research and Care (CLAHRCs): a systematic review of evaluations
Source: Implement Sci. 2018 Aug 15;13:111. doi: 10.1186/s13012-018-0805-y (PMC6094566; doi:10.1186/s13012-018-0805-y)
Supplement: Supplementary file 1 — Search strategy for CLAHRC evaluations. (DOCX 15 kb) [file 13012_2018_805_MOESM1_ESM.docx]

# **Additional File 1 Search strategy for CLAHRC evaluations**

The following databases will be searched:

- Ovid MEDLINE(R) In-Process & Other Non-Indexed Citations and Ovid MEDLINE(R) 1946 to Present
- Ovid MEDLINE(R) Epub Ahead of Print July 12, 2016
- Embase 1996 to 2016 Week 28
- PsycINFO 2002 to July Week 1 2016
- EBM Reviews - Cochrane Central Register of Controlled Trials June 2016
- EBM Reviews - Cochrane Database of Systematic Reviews 2005 to July 08, 2016
- EBM Reviews - Database of Abstracts of Reviews of Effects 1st Quarter 2016
- EBM Reviews - Cochrane Methodology Register 3rd Quarter 2012
- EBM Reviews - Health Technology Assessment 2nd Quarter 2016
- EBM Reviews - NHS Economic Evaluation Database 1st Quarter 2016
- HMIC Health Management Information Consortium 1979 to May 2016
- CINAHL
- SPORTDiscus
- Scopus
- TRiP database
- Prospero

Search strategies were modified as needed across databases. A sample strategy for MEDLINE is below.

Sample search strategy (MEDLINE (OVID)):

1. CLAHRC*.mp,in,ia.

2. ("Collaborat* for Leadership?" adj3 "Applied Health Research").mp,in,ia.

3. clarhc*.mp.

4. penclahrc.mp.

5. penclarhc*.mp.

6. ("Collaborati* for Leadership?" adj2 "research" adj2 "Applied Health").mp.

7. or/1-6

NB: mp = multi-purpose; in = institution; ia = investigator affiliation
